# Alternating Current Electroosmotic Flow of Viscoelastic Jeffreys Fluids in a pH-Regulated Slit Nanochannel

**DOI:** 10.3390/mi17070793

**Published:** 2026-06-29

**Authors:** Jiaxin Yang, Mandula Buren

**Affiliations:** School of Mathematical Science, Inner Mongolia Normal University, Hohhot 010022, China; yyyyy111220@163.com

**Keywords:** flectric double layer, AC EOF, Jeffreys fluid, pH-regulated nanochannel

## Abstract

This study investigates the electroosmotic flow (EOF) of viscoelastic Jeffreys fluids in a pH-regulated parallel-plate nanochannel, with a focus on analyzing the effects of solution pH, background salt concentration, and alternating current (AC) electric field frequency on flow characteristics. In micro- and nanoscale fluidic systems, surface charge characteristics critically govern electrokinetic flow. The surface charges in this study originate from the protonation and deprotonation reactions of silanol (SiOH) groups on the channel walls. Different from the constant surface electric potential assumed in existing studies, the surface electric potential here varies with solution pH and background salt concentration. By modulating solution pH and thereby tuning surface charge density, active and reversible control of EOF can be realized. By solving the coupled Poisson–Boltzmann equation, momentum equation, and Jeffreys constitutive equation, we obtain an analytical solution for the electric potential distribution and semi-analytical solution for the velocity field. The results show that under the chosen parameter conditions, the relaxation time *λ*_1_ enhances the velocity amplitude, while the retardation time *λ*_2_ weakens it. The EOF velocity amplitude of Jeffreys fluids is enhanced by greater pH deviation from the isoelectric point, lower ionic concentration, and higher electric field frequency. In nanochannel flows, the effect of the oscillating Reynolds number on the velocity amplitude is negligible.

## 1. Introduction

Electroosmotic flow (EOF) has attracted extensive attention owing to its broad applications in scientific research and engineering, such as micropumps, sample separation, and mixers within micro- and nanofluidic systems [1,2,3]. Among these, as core functional units, electroosmotic pumps are widely utilized in biochips and micro-total-analysis systems (µTASs) [1]. Microfluidics enables precise manipulation of fluids at the micrometer or nanometer scale. When micro- and nanochannels are in contact with an electrolyte solution, ionization of surface groups or ion adsorption induces interfacial charge, leading to the formation of an electric double layer (EDL) at the solid–liquid interface [1,2,3]. Under an external electric field, mobile ions within the EDL migrate under the Coulomb force and drive the bulk fluid through viscous drag, giving rise to EOF [4]. According to the type of applied electric field, EOF can be categorized into direct current (DC) and alternating current (AC) EOF. AC EOF has been exploited to facilitate fluid mixing via electrokinetic instability [5]. Two miscible fluids with different initial concentrations can be mixed via AC electroosmosis-driven mixing to achieve the target concentration [6].

For Newtonian fluids, EOF has been extensively studied, and its fundamental mechanisms are well established [7,8,9,10,11,12,13,14,15]. Fluid flow and mass transfer under both symmetrical and asymmetrical conditions in rectangular micro- and nanochannels have been investigated under a DC electric field [7,8] and under combined electroosmotic and pressure-driven forces [9]. Arulanandam and Li [10] and Hsu et al. [11] explored the influence of the aspect ratio on the velocity field and volumetric flow rate. Wang et al. [13] studied the flow behavior of the AC EOF of Newtonian fluids in a parallel-plate microchannel. In these studies, the surface electric potential or surface charge density was assumed to be constant. However, such an assumption is often physically unrealistic for nanochannels fabricated from pH-responsive oxides—such as SiO_2_ and Al_2_O_3_—whose surface charge density varies significantly with hydrogen ion concentration and, consequently, with the solution pH. Yeh et al. [16] analytically studied the EOF velocity, zeta potential, and surface charge density arising from surface protonation/deprotonation reactions within gated nanochannels. Tseng et al. [17] analyzed the ionic current in pH-regulated SiO_2_ nanochannels. Sadeghi et al. [18] derived infinite series solutions for electric potential, surface charge density, velocity field, and ionic conductivity in a rectangular nanochannel. Hsu and Huang [19] obtained the analytical expression for the dependence of surface electric potential on electrolyte concentration and pH. Yang et al. [20] investigated AC EOF in silica nanochannels under pH regulation, deriving an analytical solution for the electric potential and a semi-analytical solution for the velocity amplitude.

For non-Newtonian fluids, Zhao and Yang [21] systematically reviewed electrokinetic phenomena related to non-Newtonian effects in EOF, including electroosmosis, electrophoresis, and streaming potential. Choi et al. [22] and Zhao et al. [23] applied the finite element method to investigate the EOF of power-law fluids in rectangular microchannels with varying zeta potentials. Mei et al. [24] numerically investigated the EOF of a viscoelastic fluid described by the Linear Phan-Thien–Tanner (PTT) constitutive model in long nanoslits. Park and Lee [25] numerically investigated viscoelastic EOF in rectangular microchannels, showing that viscoelasticity alters the volumetric flow rate and induces secondary flows when a pressure gradient is imposed. Afonso et al. [26] derived analytical solutions for electrokinetic and pressure-driven flow of the simplified PTT model and the Finitely Extensible Nonlinear Elastic Peterlin fluid using the Debye–Hückel approximation. They found that the linear superposition principle, which holds for Newtonian fluid, is invalid for nonlinear viscoelastic fluid under combined electric and pressure potentials. The EOF and solute transport of Maxwell fluids in polyelectrolyte-grafted micro- and nanochannels under an AC electric field were investigated [27,28]. Liu et al. [29,30,31] investigated the time-periodic flows of Jeffreys and generalized Maxwell fluids in slit microchannels under electric and magnetic fields. Zhao et al. [32] presented analytical solutions for the magnetohydrodynamic flow of generalized Maxwell fluids in rectangular micropumps, analyzing the effects of Reynolds number, Hartmann number, and dimensionless relaxation time on velocity distribution. Hegde and Harikrishnan [33] numerically investigated the thermofluidics of power-law fluids considering high zeta potential and steric factor. Their study showed that slip length enhances heat transfer, whereas excessive Joule heating reverses the direction of heat flow. Kahshan et al. [34] derived an approximate analytical solution for Jeffreys fluid flow in narrow porous-walled channels. The effects of filtration coefficient, inlet pressure, and Jeffreys fluid parameters on the flow were discussed. Recently, the electrokinetic transport of non-Newtonian fluids in pH-regulated channels has attracted increasing attention. Mehta et al. [35] analytically investigated the EOF of PTT fluid in pH-sensitive microchannels with surface-charge-dependent slip. Buren et al. [36] studied Maxwell fluid behavior in pH-regulated nanochannels, showing that the fluid’s relaxation time, solution pH, salt concentration, and electric field frequency significantly influence EOF.

The Jeffreys fluid model is a generalized viscoelastic constitutive equation characterized by a relaxation time *λ*_1_ and a retardation time *λ*_2_. It naturally encompasses the Maxwell fluid (*λ*_1_ ≠ 0, *λ*_2_ = 0) and the Newtonian fluid (*λ*_1_ = 0, *λ*_2_ = 0) as limiting cases. This constitutive equation was used to describe the blood flow in a tapered artery [37,38]. The previous investigations concerning EOF in Jeffreys fluids impose the assumption that the surface electric potential is independent of the solution pH and salt concentration. Silicon, glass, and macromolecular polymers such as polymethylmethacrylate (PMMA) and polydimethylsiloxane (PDMS) are commonly used materials for micro- and nanofluidic chips. When these materials come into contact with electrolyte solutions, hydrolysis and polarization occur on the solid surface, forming silanol surface groups. These groups can carry a positive charge, a negative charge, or remain neutral, depending on the pH value of the electrolyte solution [1,2,18]. Up to now, no studies have investigated the AC EOF of viscoelastic Jeffreys fluid in pH-regulated slit nanochannels under no-slip boundary conditions. Motivated by this gap, we select the Jeffreys fluid model as a constitutive equation to investigate the AC EOF of viscoelastic fluid in a pH-regulated parallel-plate nanochannel. The remainder of this paper is organized as follows. Section 2 presents the physical model and governing equations and derives their analytical solutions. Section 3 provides a detailed analysis and discussion of the parametric effects on AC EOF. Section 4 summarizes the main findings and conclusions.

## 2. Mathematical Model

### 2.1. Electric Potential Distribution

**Figure 1:**
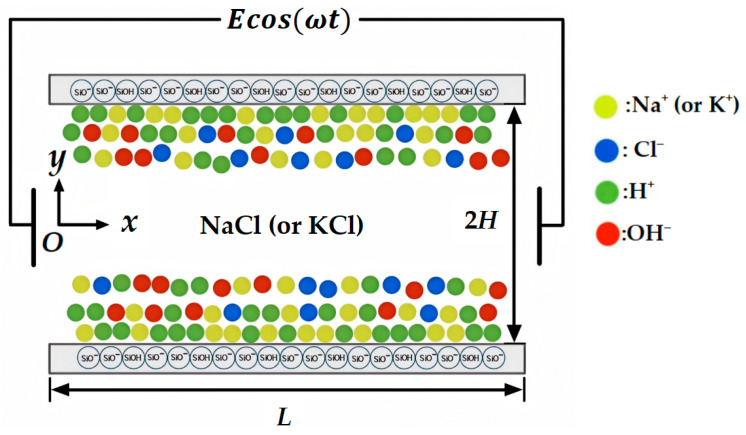
Schematic of alternating current electroosmotic flow in a slit silica nanochannel.

As shown in Figure 1, this study analyzes the AC EOF behavior of a Jeffreys fluid within a pH-regulated parallel-plate silica nanochannel. A Cartesian coordinate system (*x*, *y*) is used, with the origin set at the center of the nanochannel. The nanochannel has a height of 2*H*, length *L*, and width *W*, with the condition that the channel height is much smaller than the length and width, i.e., (2*H* << *L*, *W*). In the parallel-plate channel, we assume that the flow field is independent of the variable *z*, namely, $\frac{\partial }{\partial z}=0$. The background salt is NaCl (or KCl). The pH is regulated by HCl and NaOH (or KOH) [17,18]. At the interface between the channel wall and the solution, a chemical reaction occurs between silica and water to form silicon hydroxyl (SiOH) groups. These functional groups then undergo protonation and deprotonation reactions with hydrogen ions in the solution, thereby generating surface charges on the nanochannel wall [17,18]:

(1) $$SiOH+H^{+}\overset{K_{+}}{↔}{SiOH}_{2}^{+},SiOH\overset{K_{-}}{↔}{SiO}^{-}+H^{+}.$$

$K_{+}$ and $K_{-}$ are the equilibrium constants for the protonation and deprotonation reactions [17,18,20],

(2) $$K_{+}=\frac{\Gamma_{{SiOH}_{2}^{+}}}{\Gamma_{SiOH}{\left[H^{+}\right]}_{s}},K_{-}=\frac{\Gamma_{{SiO}^{-}}{\left[H^{+}\right]}_{s}}{\Gamma_{SiOH}},$$

where $\Gamma_{{SiO}^{-}}$, $\Gamma_{{SiOH}_{2}^{+}}$ and $\Gamma_{SiOH}$ represent the surface site density of ${SiO}^{-}$, ${SiOH}_{2}^{+}$ and SiOH, respectively; ${\left[H^{+}\right]}_{s}$ represents the ion concentration of $H^{+}$ at the surface of the channel, ${\left[H^{+}\right]}_{s}=10^{-pH}exp\left(-\frac{F\psi_{s}}{RT}\right)$; $10^{-pH}$ denotes the bulk molar concentrations of $H^{+}$; $\psi_{s}$ is the surface electric potential of the channel; *F* is the Faraday constant; *R* is the gas constant; and *T* is the absolute temperature.

The surface charge density of the channel $\sigma_{s}$ is [17,18,20]

(3) $$\sigma_{s}=10^{18}e\left(\Gamma_{{SiOH}_{2}^{+}}-\Gamma_{{SiO}^{-}}\right)=-\frac{10^{18}F\Gamma_{t}}{N_{A}}\left(\frac{K_{-}-K_{+}{\left[H^{+}\right]}_{s}^{2}}{K_{-}+{\left[H^{+}\right]}_{s}+K_{+}{\left[H^{+}\right]}_{s}^{2}}\right).$$

Here, $\Gamma_{t}=\Gamma_{{SiOH}_{2}^{+}}+\Gamma_{{SiO}^{-}}+\Gamma_{SiOH}$ denotes the total site density; *e* denotes the elementary charge; and *N_A_* denotes Avogadro’s constant.

At a thermodynamic equilibrium state [2], the concentrations of Na^+^ (or K^+^), Cl^−^, H^+^ and OH^−^ in the solution obey the Boltzmann distributions $C_{i}=C_{i0}exp\left(-\frac{z_{i}F\psi }{RT}\right)$ (*i* = 1, 2, 3, 4), respectively. Here, $C_{i0}$ is the bulk concentration of these ions, and the electric potential *ψ* is set to zero at *y* = 0. *z_i_* denotes the ionic valence, and *z*_1_ = *z*_3_ = −*z*_2_ = −*z*_4_ = 1 [17,18,20].

Based on the electric neutrality of the solution, it is known that *C*_0_ = *C*_10_ + *C*_30_ = *C*_20_ + *C*_40_. When pH ≤ 7, the solution’s pH is adjusted with HCl: *C*_10_ = *C_M_* × 10^3^, *C*_20_ = *C*_10_ + 10^−pH+3^ −10^pH−14+3^, *C*_30_ = 10^−pH+3^, and *C*_40_ = 10^pH−14+3^. When pH > 7, the solution’s pH is adjusted with NaOH (or KOH): *C*_10_ = *C*_20_ − 10^pH+3^ + 10^pH−14+3^, *C*_20_ = *C_M_* × 10^3^, *C*_30_ = 10^pH+3^, and *C*_40_ = 10^pH−14+3^. *C_M_* represents the molar concentration of NaCl (or KCl) [17,18,20].

According to the theory of electrostatics, the electric potential ψ within the EDL in the homogeneous parallel-plate channel satisfies the one-dimensional Poisson–Boltzmann equation [1,29]:

(4) $$\frac{d^{2}\psi }{{dy}^{2}}=-\frac{\rho_{e}}{\varepsilon },$$

where the volumetric charge density $\rho_{e}$ in the solution is described by

(5) $$\rho_{e}=F\sum_{i=1}^{4}z_{i}C_{i}=-2FC_{0}\sinh\frac{F\psi }{RT},$$

where ε represents the dielectric constant of the solution. The electric potential equals the surface electric potential at the boundary and is symmetric about the mid-plane between the two surfaces, i.e.,

(6) $${\psi |}_{y=H}=\psi_{s},\frac{d\psi }{dy}{|}_{y=0}=0.$$

Due to electroneutrality, the surface charge is balanced by the charge in the solution. This leads to

(7) $$\sigma_{s}+\int_{0}^{H}\rho_{e}dy=0.$$

Substituting Equation (4) into this relation gives

(8) $$\sigma_{s}=\varepsilon \int_{0}^{H}\frac{d^{2}\psi }{{dy}^{2}}dy=\varepsilon \frac{d\psi }{dy}{|}_{y=H}.$$

Substituting Equation (5) into Equation (4) and integrating with respect to *y* from 0 to *H* yields

(9) $$\frac{d\psi }{dy}{|}_{y=H}=\sqrt{\frac{8RTC_{0}}{\varepsilon }}\sinh\left(\frac{F\psi_{s}}{2RT}\right).$$

The surface charge density $\sigma_{s}$ is equal to

(10) $$\sigma_{s}=\varepsilon \frac{d\psi }{dy}{|}_{y=H}=\sqrt{8RTC_{0}\varepsilon }\sinh\left(\frac{F\psi_{s}}{2RT}\right).$$

Dimensionless parameters are introduced

(11) $$y^{*}=\frac{y}{H},K=\frac{H}{\lambda_{D}},\psi^{*}=\frac{\psi }{\psi_{0}},\psi_{0}=\frac{RT}{F},\lambda_{D}=\sqrt{\frac{\varepsilon RT}{2C_{0}F^{2}}},$$

where *K* is called the dimensionless electrokinetic width.

By combining Equations (3) and (10) and substituting the dimensionless parameters, we obtain

(12) $$\sigma_{s}=-\sigma_{0}\left(\frac{K_{-}-K_{+}{\left[H^{+}\right]}_{s}^{2}}{K_{-}+{\left[H^{+}\right]}_{s}+K_{+}{\left[H^{+}\right]}_{s}^{2}}\right)=\frac{2\varepsilon \psi_{0}K}{H}\sinh\left(\frac{\psi_{s}^{*}}{2}\right),$$

where $\sigma_{0}=\frac{10^{18}F\Gamma_{t}}{N_{A}}$. Under a given protonation equilibrium constant, the surface charge density is determined by the surface proton concentration, while the surface proton concentration is related to the surface electric potential through the Boltzmann distribution. The dimensionless surface electric potential can be obtained numerically from Equation (12) using the fzero function in MATLAB R2022a, provided that all other parameters are specified.

Substituting Equations (5) and (11) into Equations (4) and (6), we get

(13) $$\frac{d^{2}\psi^{*}}{{dy^{*}}^{2}}=K^{2}\sinh\psi^{*},$$

(14) $${\psi^{*}|}_{y^{*}=1}=\psi_{s}^{*},\frac{{d\psi }^{*}}{{dy}^{*}}{|}_{y^{*}=0}=0.$$

Solving Equation (13) subject to the boundary conditions (14) yields the analytical expression for the dimensionless electric potential. The detailed derivations are given in Appendix A.

(15) $$\psi^{*}=4\tanh^{-1}\left[exp(K(y^{*}-1))\tanh\left(\frac{\psi_{s}^{*}}{4}\right)\right].$$

### 2.2. Velocity Distribution

Under the action of an electric field $\overset{⃑}{E}$, the continuity equation and the Cauchy momentum equation for an incompressible electrolyte solution are, respectively, given by

(16) $$\nabla \cdot \overset{⃑}{u}=0,$$

(17) $$\rho \left[\frac{\partial \overset{⃑}{u}}{\partial t}+\left(\overset{⃑}{u}\cdot \nabla \right)\overset{⃑}{u}\right]=\nabla \cdot \overset{⃑}{\tau }+\rho_{e}\overset{⃑}{E}.$$

Here, no pressure difference is applied across the channel; hence, the pressure gradient term is neglected.

As shown in Figure 1, when an AC electric field $\overset{⃑}{E}=(Ecos(\omega t),0,0)$ is imposed along the *x*-direction, it exerts a Coulomb force to the net charge densities, thereby generating a unidirectional flow with velocity components (*u*,0,0). Here, we assume that the velocity components in the *y*- and *z*-directions are zero. Due to the assumption that the flow field is independent of the variable *z*, the continuity equation becomes ∂u/∂x=0, implying that the axial velocity component *u* is independent of the variable *x* and depends only on the variable *y* and time *t*.

Based on these assumptions, the momentum equation in the *x*-direction reduces to

(18) $$\rho \frac{\partial u}{\partial t}=\frac{\partial }{\partial y}\tau_{yx}+\rho_{e}Ecos\left(\omega t\right).$$

Here, ρ denotes the fluid density. The constitutive equation of a Jeffreys fluid is given by [29]:

(19) $$(1+\lambda_{1}\frac{\partial }{\partial t})\tau_{yx}=\eta (1+\lambda_{2}\frac{\partial }{\partial t})\frac{\partial u}{\partial y}.$$

Here, *τ_yx_* denotes the shear stress in the *x*-direction on the *y*-plane; *λ*_1_, *λ*_2_, and *η* denote the relaxation time, retardation time, and the zero shear rate viscosity.

Due to the hydrophilic nature of the silica surface, the fluid obeys the no-slip boundary condition at the walls [20]:

(20) $${u|}_{y=H}=0,\frac{\partial u}{\partial y}{|}_{y=0}=0.$$

Here, the second one is due to the symmetry of the velocity distribution.

From Equations (18) and (19), we obtain

(21) $$\rho \frac{\partial u}{\partial t}+\lambda_{1}\rho \frac{\partial^{2}u}{\partial t^{2}}=\eta \frac{\partial^{2}u}{\partial y^{2}}+\eta \lambda_{2}\frac{\partial^{3}u}{\partial y^{2}\partial t}+\rho_{e}Ecos\omega t+{\lambda_{1}\rho }_{e}\frac{\partial }{\partial t}(Ecos\omega t).$$

The oscillatory velocity field induced by the AC electric field can be expressed in a complex form

(22) $$u=Re\{U\left(y\right)e^{i\omega t}\},Ecos\omega t=Re\{Ee^{i\omega t}\},$$

where Re{ } denotes the real part of the complex number and *i* denotes the imaginary unit.

Substituting Equation (22) into Equation (21), we obtain

(23) $$\eta \left(1+i\omega \lambda_{2}\right)\frac{d^{2}U}{dy^{2}}-\left(i\omega -\omega^{2}\lambda_{1}\right)\rho U=-\rho_{e}E(1+i\omega \lambda_{1}).$$

Introduce the following dimensionless variables

(24) $$U^{*}\left(y^{*}\right)=\frac{U}{U_{eo}},U_{eo}=-\frac{\varepsilon ERT}{\eta F},{Re}_{\omega }=\frac{H^{2}\omega \rho }{\eta },$$

where Re*_ω_* denotes the oscillating Reynolds number.

Substituting Equations (5), (11) and (24) into Equation (23) and simplifying yields a second-order linear nonhomogeneous ordinary differential equation:

(25) $$\frac{d^{2}U^{*}}{d{y^{*}}^{2}}-\frac{i-\omega \lambda_{1}}{1+i\omega \lambda_{2}}{Re}_{\omega }U^{*}=-\frac{1+i\omega \lambda_{1}}{1+i\omega \lambda_{2}}K^{2}sinh(\psi^{*}).$$

From Equations (20), (21) and (23), we obtain

(26) $${U^{*}|}_{y^{*}=1}=0,\frac{{dU}^{*}}{{dy}^{*}}{|}_{y^{*}=0}=0.$$

Let $\alpha^{2}=\frac{i-\omega \lambda_{1}}{1+i\omega \lambda_{2}}{Re}_{\omega }$, $S\left(y^{*}\right)=-\frac{1+i\omega \lambda_{1}}{1+i\omega \lambda_{2}}K^{2}sinh(\psi^{*})$. The solution of Equation (25) can be expressed as

(27) $$U^{*}=U_{c}^{*}+U_{p}^{*}.$$

The general solution of the corresponding homogeneous equation is

(28) $$U_{c}^{*}=C_{1}e^{\alpha y^{*}}+C_{2}e^{-\alpha y^{*}}.$$

The particular solution of the nonhomogeneous Equation (25) is

(29) $$U_{p}^{*}=D_{1}(y^{*})e^{\alpha y^{*}}+D_{2}(y^{*})e^{-\alpha y^{*}}.$$

Using the method of variation of parameters, we set

(30) $$\frac{dD_{1}}{dy^{*}}e^{\alpha y^{*}}+\frac{dD_{2}}{dy^{*}}e^{-\alpha y^{*}}=0.$$

(31) $$\alpha (\frac{dD_{1}}{dy^{*}}e^{\alpha y^{*}}-\frac{dD_{2}}{dy^{*}}e^{-\alpha y^{*}})=S\left(y^{*}\right).$$

Solving, we find

(32) $$D_{1}(y^{*})=\frac{1}{2\alpha }\int_{0}^{y^{*}}S\left(\tilde{y}\right)e^{-\alpha \tilde{y}}d\tilde{y},\;D_{2}(y^{*})=-\frac{1}{2\alpha }\int_{0}^{y^{*}}S\left(\tilde{y}\right)e^{\alpha \tilde{y}}d\tilde{y}.$$

Substituting Equations (27)–(29) and (32) into the boundary condition (26), we obtain

(33) $$C_{1}=C_{2}=\frac{A}{2},$$

where

(34) $$A=-\frac{\left(1+i\omega \lambda_{1}\right)K^{2}}{\sqrt{\left(i-\omega \lambda_{1}\right)\left(1+i\omega \lambda_{2}\right){Re}_{\omega }}cosh\sqrt{\frac{i-\omega \lambda_{1}}{1+i\omega \lambda_{2}}{Re}_{\omega }}}f(1),$$

(35) $$f\left(y^{*}\right)=\int_{0}^{y^{*}}sinh{[\psi }^{*}(\tilde{y})]sinh[\sqrt{\frac{i-\omega \lambda_{1}}{1+i\omega \lambda_{2}}{Re}_{\omega }}(\tilde{y}-y^{*})]d\tilde{y}.$$

Combining Equations (27)–(29) and (33), we obtain the solution for the velocity amplitude

(36) $$U^{*}\left(y^{*}\right)=Acosh(\sqrt{\frac{i-\omega \lambda_{1}}{1+i\omega \lambda_{2}}{Re}_{\omega }}y^{*})+\frac{(1+i\omega \lambda_{1})K^{2}}{\sqrt{(i-\omega \lambda_{1})(1+i\omega \lambda_{2}){Re}_{\omega }}}f\left(y^{*}\right).$$

When *λ*_1_ ≠ 0, *λ*_2_ = 0, Equation (36) becomes the velocity amplitude of the AC EOF of a Maxwell fluid [36]. When *λ*_1_ = *λ*_2_ = 0, Equation (36) becomes the velocity amplitude of the AC EOF of a Newtonian fluid [20].

## 3. Results and Discussion

In this study, the AC EOF of a Jeffreys fluid in a pH-regulated parallel-plate nanochannel is investigated theoretically. Different from existing research concerning the EOF behavior of Jeffreys fluids in channels under constant surface charge density or constant surface electric potential boundary conditions, the surface electric potential in this study is dependent on the solution pH and the bulk ionic concentration. Particular attention is paid to the combined effects of the relaxation time (*λ*_1_) and retardation time (*λ*_2_) of the Jeffreys fluid, the background salt concentration (*C_M_*), and the solution pH on the electric potential distribution and velocity amplitude. In the subsequent discussion, the following parameter ranges are chosen based on the existing research [8,17,18,20,27]: *ρ* = 1000 kg·m^−3^, *η* = 0.001 kg·m^−1^·s^−1^, *H* = 50 × 10^−9^ m, *T* = 300 K, *R* = 8.314 J·mol^−1^·K^−1^, *ε* = 80 × 8.854 × 10^−12^ F·m^−1^, Γ*_t_* = 6 nm^−2^, *N_A_* = 6.022 × 10^23^ mol^−1^, *F* = 96,485.33 C·mol^−1^, *C_M_* ~ 0.002–0.1 kmol/m^3^, pH ~ 2–10, *ω* ~ 0–10^3^ s^−1^, p*K*_−_ = 8, p*K_+_* = 1.9 (p*K*_±_ = −log *K*_±_), *λ*_1_ ~ 0–0.06 s, *λ*_2_ ~ 0–0.05 s. To guarantee the validity of the undisturbed EDL assumption, *λ*_1_ is less than 2π/*ω.* Moreover, *λ*_1_ is typically greater than *λ*_2_. Therefore, *λ*_2_*ω* < *λ*_1_*ω* < 2π [29]. The dimensionless electrokinetic width *K* = *H*/*λ_D_* ~ 7–51.32, indicating that the EDLs remain non-overlapping. Re*_ω_* ~ 0–2.5 × 10^−6^. The influence of Re*_ω_* on the velocity amplitude can be neglected.

**Figure 2:**
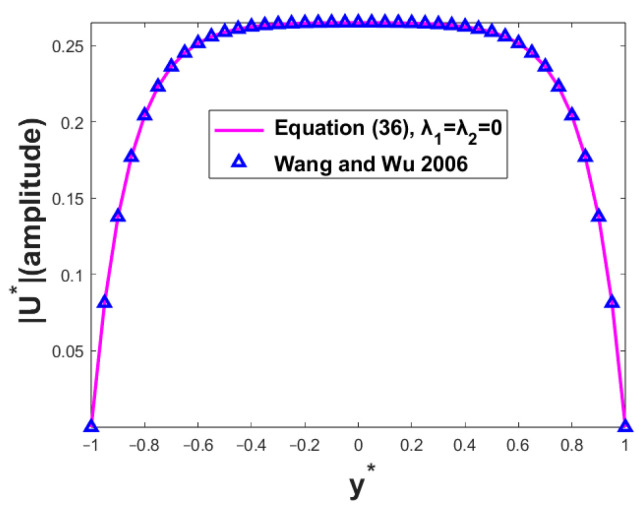
Comparison of the velocity amplitude given by Equation (36) with *λ*_1_ = *λ*_2_ = 0 and that obtained by Wang and Wu [13].

When *C_M_* = 0.002 kmol/m^3^, pH = 5, and *ω* = 10^2^ Hz, we obtain *K* = 7.28, Re*_ω_* = 2.5 × 10^−7^, $\psi_{s}^{*}$ ≈ −0.266. Figure 2 compares the velocity amplitude of Newtonian AC EOF—computed from Equation (36) with *λ*_1_ = *λ*_2_ = 0 in the present study—with that of the Newtonian AC EOF reported by Wang and Wu [13] under identical dimensionless parameters (*K* = 7.28, Re*_ω_* = 2.5 × 10^−7^, $\psi_{s}^{*}$ ≈ −0.266). The two curves are in perfect agreement.

**Figure 3:**
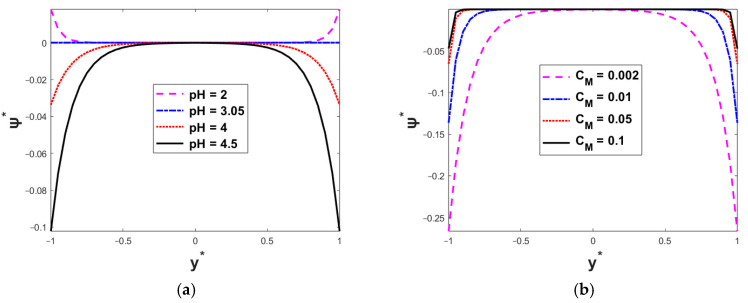
Dimensionless electric potential for various pH values and *C_M_*: (**a**) *C_M_* = 0.002 kmol/m^3^; (**b**) pH = 5.

Figure 3a illustrates the electric potential distribution, which is symmetrically distributed about the channel centerline. When the pH values are 2, 4, and 4.5, the corresponding *K* values are 17.78, 7.44, and 7.32, respectively. It is found that as pH increases, *K* decreases and the thickness of the EDL increases. As the pH value gradually deviates from the isoelectric point (pH = 3.05), the absolute value of the surface electric potential increases accordingly, thereby enhancing the driving force for EOF. The surface electric potential equals zero at pH = 3.05, leading to zero surface charge density on the channel wall. Figure 3b shows the effect of different *C_M_* on the electric potential distribution at a fixed pH of 5. As *C_M_* increases, the *K* rises from 7.28 to 51.32, while the EDL thickness decreases correspondingly. Moreover, the absolute value of the surface electric potential decreases, resulting in a weaker electroosmotic driving force.

**Figure 4:**
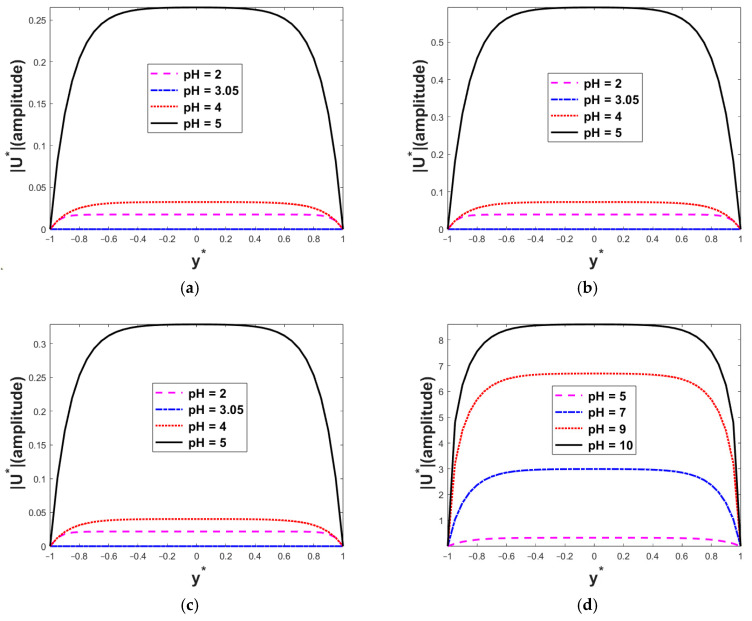
Dimensionless velocity amplitude at *C_M_* = 0.002 kmol/m^3^ and *ω* = 10^2^ Hz for different values of pH, *λ*_1_, and *λ*_2_: (**a**) *λ*_1_ = *λ*_2_ = 0 s; (**b**) *λ*_1_ = 0.02 s, *λ*_2_ = 0 s; (**c**,**d**) *λ*_1_ = 0.02 s, *λ*_2_ = 0.015 s.

Figure 4 shows the variation in the velocity amplitude with pH for different fluid types. At the pH values of 2, 4, 5, 7, 9, and 10, the corresponding *K* values are 17.78, 7.44, 7.28, 7.26, 7.28, and 7.44, respectively. Notably, the *K* values at pH = 4 and pH = 10 are identical, as are those at pH = 5 and pH = 9. This is because when pH is symmetric about pH = 7, *C*_0_ remains unchanged, resulting in the same *λ_D_* and, subsequently, the same *K* value. Therefore, the EDL thickness is the same at these pH values. *K* is related to the shape of the velocity profile. When *K* decreases, the velocity profile becomes less plug-like. The magnitude of the velocity amplitude is related to pH. At pH = 3.05, both the surface electric potential and the surface charge density are zero. Consequently, no EOF occurs, leading to a velocity amplitude of zero. As the pH deviates from this value, the velocity amplitude becomes non-zero, and the greater the deviation, the higher the flow velocity. The reason for this is that a lower H^+^ concentration (higher pH) promotes the dissociation of silanol groups (SiOH) into negatively charged SiO^−^ groups, which increases the net surface charge, thereby raising the absolute value of surface electric potential with increasing solution pH (see Figure 3). The greater the surface electric potential is, the greater the EOF will be. Compared to the Newtonian fluid (*λ*_1_ = *λ*_2_ = 0), the Maxwell fluid (*λ*_1_ ≠ 0, *λ*_2_ = 0) shows the most pronounced enhancement in velocity amplitude, while the Jeffreys fluid (*λ*_1_ ≠ 0, *λ*_2_ ≠ 0) exhibits an intermediate increase. This indicates that *λ*_1_ enhances the EOF velocity amplitude, whereas *λ*_2_ suppresses it. Physically, the stress relaxation time *λ*_1_ enhances flow velocity via elastic behavior at certain frequencies. The strain retardation time *λ*_2_ characterizes the time lag of fluid deformation under applied shear stress. It introduces a damping effect and generally suppresses the AC EOF velocity.

**Figure 5:**
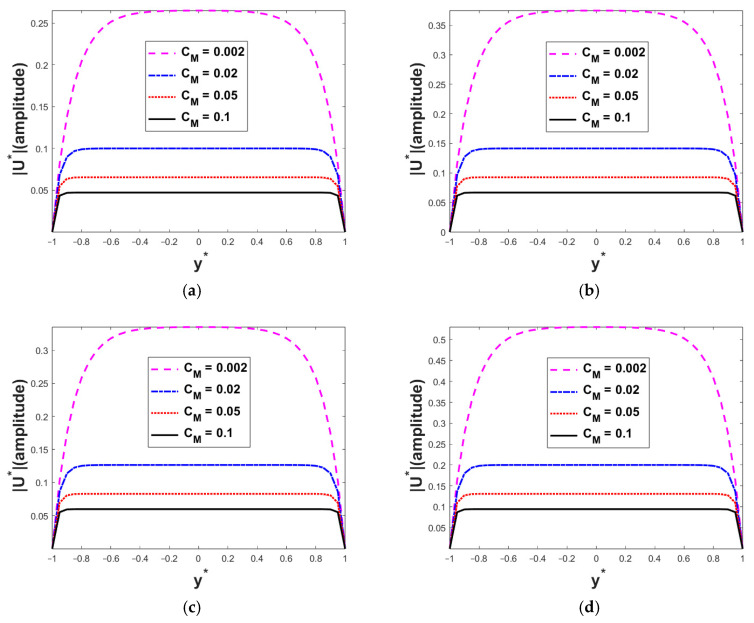
Dimensionless velocity amplitude at pH = 5 and *ω* = 10^2^ Hz for different values of *C_M_*, *λ*_1_, and *λ*_2_: (**a**) *λ*_1_ = *λ*_2_ = 0 s; (**b**) *λ*_1_ = 0.01 s, *λ*_2_ = 0 s; (**c**) *λ*_1_ = 0.01 s, *λ*_2_ = 0.005 s; and (**d**) *λ*_1_ = 0.02 s, *λ*_2_ = 0.005 s.

Figure 5 shows the variation in the velocity amplitude with *C_M_* for different fluid types. At the *C_M_* values of 0.002, 0.02, 0.05, and 0.1 kmol/m^3^, the corresponding *K* values are 7.28, 22.96, 36.29, and 51.32, respectively. Therefore, as *C_M_* increases, *K* increases, while the thickness of the EDL decreases and the velocity profile becomes more plug-like. In each subplot, as *C_M_* increases, the velocity amplitude continuously decreases. Physically, the surface electric potential drops with the rising salt concentration. A higher ionic concentration induces more counterion adsorption on the channel surface, which diminishes net effective charge and thereby decreases the surface electric potential. The smaller the surface electric potential is, the smaller the EOF will be. From Figure 5, the velocity amplitude decreases monotonically with increasing *C_M_* in all cases. This trend is most gradual for the Newtonian fluid (*λ*_1_ = *λ*_2_ = 0), while it becomes significantly more pronounced for the Maxwell fluid (*λ*_1_ ≠ 0, *λ*_2_ = 0). For the Jeffreys fluid (*λ*_1_ ≠ 0, *λ*_2_ ≠ 0), the decrease is intermediate, and the effect of *C_M_* is amplified as *λ*_1_ increases. Physically, higher *C_M_* compresses the EDL and reduces the absolute surface potential, weakening the EOF driving force and, thus, lowering the velocity amplitude.

**Figure 6:**
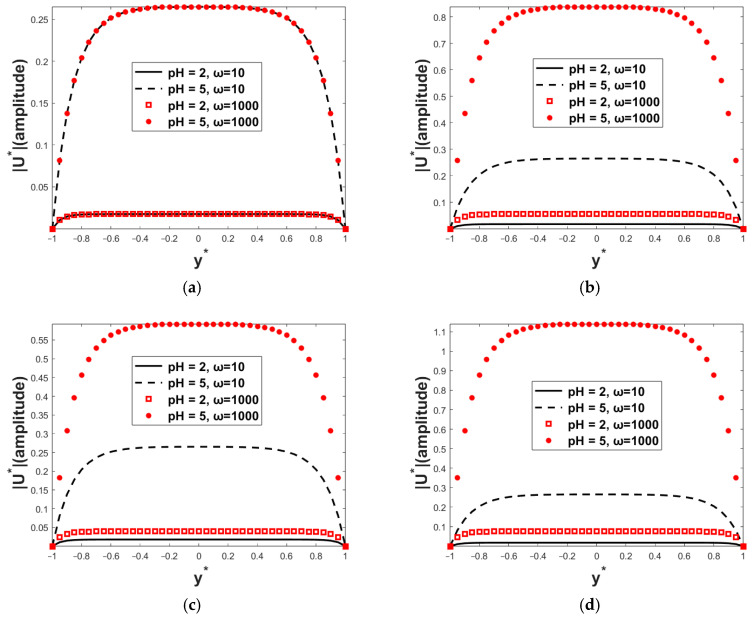
Dimensionless velocity amplitude at *C_M_* = 0.002 kmol/m^3^ for different values of pH, *ω*, *λ*_1_, and *λ*_2_: (**a**) *λ*_1_ = *λ*_2_ = 0 s; (**b**) *λ*_1_ = 0.003 s, *λ*_2_ = 0 s; (**c**) *λ*_1_ = 0.003 s, *λ*_2_ = 0.001 s; and (**d**) *λ*_1_ = 0.006 s, *λ*_2_ = 0.001 s.

Figure 6 illustrates the influence of pH and *ω* on the fluid velocity amplitude for various fluid types. When the pH values are 2 and 5, the corresponding *K* values are 17.78 and 7.28, respectively. Figure 6a shows that the velocity amplitude curves of the Newtonian fluid (λ_1_ = *λ*_2_ = 0) at *ω* = 1000 and *ω* = 10 are superimposed at the same pH, indicating that the frequency *ω* has no influence on the velocity amplitude of the Newtonian fluid within the investigated parameter range. This observation is consistent with Equation (25), which becomes independent of the *λ*_1_*ω* and *λ*_2_*ω* terms when *λ*_1_ = *λ*_2_ = 0. Furthermore, the oscillating Reynolds number remains sufficiently small at both frequencies: Re*_ω_* = 2.5 × 10^−8^ at *ω* = 10 and Re*_ω_* = 2.5 × 10^−6^ at *ω* = 1000. Thus, it does not significantly affect momentum diffusion near the wall. In Figure 6b (Maxwell fluid, *λ*_1_ ≠ 0, *λ*_2_ = 0) and Figure 6c,d (Jeffreys fluids, *λ*_1_ ≠ 0, *λ*_2_ ≠ 0), the frequency *ω* exhibits a promoting effect on the velocity amplitude: at the same pH, the velocity amplitude for *ω* = 1000 is significantly higher than that for *ω* = 10. The promoting effect of *ω* increases with *λ*_1_, which arises from the term *λ*_1_*ω* in Equation (25).

**Figure 7:**
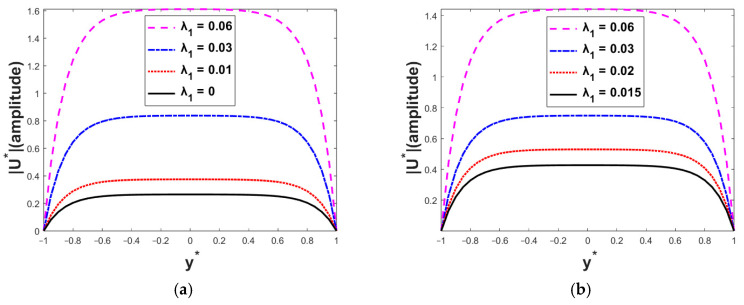

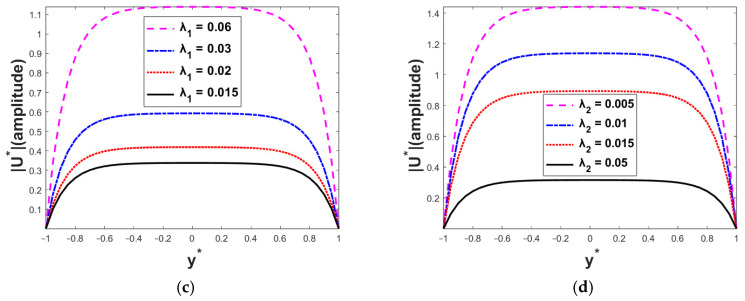
Dimensionless velocity amplitude at *C_M_* = 0.002 kmol/m^3^, pH = 5, *K* = 7.28 and *ω* = 10^2^ Hz for different values of *λ*_1_ and *λ*_2_: (**a**) *λ*_2_ = 0 s; (**b**) *λ*_2_ = 0.005 s; (**c**) *λ*_2_ = 0.01 s; and (**d**) *λ*_1_ = 0.06 s.

As shown in Figure 7a–c, the dimensionless velocity amplitude increases significantly with the relaxation time *λ*_1_. A larger *λ*_1_ enhances the elastic behavior of the fluid, which reduces the effective viscosity and flow resistance, leading to a higher AC EOF velocity at certain frequencies. Figure 7d shows that the velocity amplitude decreases monotonically with increasing retardation time *λ*_2_. The strain retardation effect introduces additional damping to the flow, weakening the elastic enhancement caused by *λ*_1_ and suppressing the AC EOF velocity.

## 4. Conclusions

This work conducts an analytical study on the AC EOF of a Jeffreys fluid in pH-regulated parallel-plate silica nanochannels. The surface charge on the channel wall originates from the protonation and deprotonation of the silanol (SiOH) functional group. By solving the coupled Poisson–Boltzmann equation, Cauchy momentum equation, and Jeffreys constitutive equation, the solutions for the electric potential distribution and velocity field are obtained analytically. The main conclusions are as follows: distinct from existing work on the AC EOF of Jeffreys fluids under constant surface charge distribution or constant surface electric potential, the surface and internal channel electric potentials depend on pH and bulk salt concentration, changing ionic concentrations in EDL to alter the AC EOF of the Jeffreys fluid; within the investigated parameter range, the relaxation time *λ*_1_ enhances EOF, whereas the retardation time *λ*_2_ suppresses it, revealing opposite effects that distinguish Jeffreys fluids from Newtonian and Maxwell fluids. Both a large deviation of the solution pH from the isoelectric point and a low salt concentration are key conditions for enhancing EOF, as both factors increase the net electric body force acting on the fluid; increasing the frequency of electric field can amplify the velocity amplitude of the EOF of Jeffreys fluids.

## Data Availability

The data presented in this study is available on request from the corresponding author.

## Nomenclature

The following nomenclature are used in this manuscript:

$C_{i}$	Concentration of the *i*th ionic species (mol·m^−3^)
$C_{i0}$	Bulk concentration of the *i*th ionic species (mol·m^−3^)
$C_{M}$	Background salt concentration (kmol·m^−3^)
*e*	Elementary charge (C)
*E*	Electric field (V·m^−1^)
*F*	Faraday constant (C·mol^−1^)
*H*	Half nanochannel height (m)
${\left[H^{+}\right]}_{s}$	$\mathrm{Concentration}\;\mathrm{of}\;H^{+}$ at the surface of the channel (kmol·m^−3^)
*K*	Dimensionless electrokinetic width
$K_{+}$	Equilibrium constant for the protonation reaction (kmol·m^−3^)^−1^
$K_{-}$	Equilibrium constant for the deprotonation reaction (kmol·m^−3^)
*L*	Nanochannel length (m)
*N_A_*	Avogadro’s constant (mol^−1^)
*R*	Gas constant (J·mol^−1^·K^−1^)
${Re}_{\omega }$	Oscillating Reynolds number
*T*	Absolute temperature (K)
u	Velocity component (m·s^−1^)
U	Complex velocity amplitude (m·s^−1^)
$U^{*}$	Dimensionless velocity amplitude
*W*	Nanochannel width (m)
*y*	Coordinates (m)
$y^{*}$	Dimensionless coordinate
*z_i_*	Valence of the *i*th ionic species
(*x*, *y*)	Cartesian coordinate system
$\Gamma_{{SiO}^{-}}$	$\mathrm{Surface}\;\mathrm{site}\;\mathrm{density}\;\mathrm{of}\;{SiO}^{-}$(nm^−2^)
$\Gamma_{{SiOH}_{2}^{+}}$	$\mathrm{Surface}\;\mathrm{site}\;\mathrm{density}\;\mathrm{of}\;{SiOH}_{2}^{+}$(nm^−2^)
$\Gamma_{SiOH}$	Surface site density of SiOH(nm^−2^)
$\Gamma_{t}$	Total site density of functional groups (nm^−2^)
ε	Dielectric constant of the solution (F·m^−1^)
*η*	Zero shear rate viscosity (kg·m^−1^·s^−1^)
*λ*_1_	Relaxation time (s)
*λ*_2_	Retardation time (s)
*λ_D_*	Debye length (m)
*ρ*	Fluid density (kg·m^−3^)
$\rho_{e}$	Volumetric charge density (C·m^−3^)
$\sigma_{s}$	Surface charge density of the channel (C·m^−2^)
*τ_yx_*	Shear stress in the *x*-direction on the *y*-plane (N·m^−2^)
ψ	Electric potential (V)
$\psi^{*}$	Dimensionless electric potential
$\psi_{s}$	Surface electric potential of the channel (V)
$\psi_{s}^{*}$	Dimensionless surface electric potential of the channel
ω	Frequency of electric field (s^−1^)

## Abbreviations

The following abbreviations are used in this manuscript:

EOF	Electroosmotic flow
EDL	Electric double layer
AC	Alternating current
DC	Direct current

## Appendix A. Analytical Solution of Poisson–Boltzmann Equation

Starting from the dimensionless Poisson–Boltzmann equation:

(A1) $$\frac{d^{2}\psi^{*}}{{dy^{*}}^{2}}=K^{2}\sinh\psi^{*},$$

subject to the boundary conditions:

(A2) $${\psi^{*}|}_{y^{*}=1}=\psi_{s}^{*},\frac{{d\psi }^{*}}{{dy}^{*}}{|}_{y^{*}=0}=0.$$

Multiplying both sides of Equation (A1) by $2\frac{d\psi^{*}}{dy^{*}}$ yields:

(A3) $$2\frac{d\psi^{*}}{dy^{*}}\frac{d^{2}\psi^{*}}{{dy^{*}}^{2}}=2K^{2}\frac{d\psi^{*}}{dy^{*}}\sinh\psi^{*}.$$

Equation (A3) may be rewritten as

(A4) $$\frac{d}{dy^{*}}{\left(\frac{d\psi^{*}}{dy^{*}}\right)}^{2}=2K^{2}\frac{d}{dy^{*}}(\cosh\psi^{*}).$$

Integrating with respect to $y^{*}$, we obtain

(A5) $${\left(\frac{d\psi^{*}}{dy^{*}}\right)}^{2}=2K^{2}\cosh\psi^{*}+C_{1}.$$

Substituting the condition $\psi^{*}\left(0\right)=0$ and the symmetry condition $\frac{{d\psi }^{*}}{{dy}^{*}}{|}_{y^{*}=0}=0$ into Equation (A5) gives $C_{1}=-2K^{2}$; hence,

(A6) $${\left(\frac{d\psi^{*}}{dy^{*}}\right)}^{2}=2K^{2}(\cosh\psi^{*}-1).$$

Using the identity $\cosh\psi^{*}-1=2\sinh^{2}\left(\frac{\psi^{*}}{2}\right)$ and taking the square root, we obtain

(A7) $$\frac{d\psi^{*}}{dy^{*}}=2K\sinh\frac{\psi^{*}}{2}.$$

Integrating with respect to $y^{*}$, we obtain

(A8) $$\tanh\frac{\psi^{*}}{4}=C_{4}e^{Ky^{*}}.$$

Applying the boundary condition $\psi^{*}(1)=\psi_{s}^{*}$ yields $C_{4}=e^{-K}\tanh\frac{\psi_{s}^{*}}{4}$. Substituting this result back into Equation (A8):

(A9) $$\tanh\frac{\psi^{*}}{4}=e^{-K}\tanh\frac{\psi_{s}^{*}}{4}\cdot e^{Ky^{*}}=e^{K(y^{*}-1)}\tanh\frac{\psi_{s}^{*}}{4}.$$

Taking the inverse hyperbolic tangent yields

(A10) $$\psi^{*}=4\tanh^{-1}\left[exp(K\left(y^{*}-1\right))\tanh\frac{\psi_{s}^{*}}{4}\right].$$

This is exactly Equation (15) in the main text.
